# Melatonin as a potential adjuvant to mitigate depakine‑induced testicular damage in rats through its biological features

**DOI:** 10.1186/s12906-025-04969-w

**Published:** 2025-06-25

**Authors:** Maggie E. Amer, Mohamed A. Dardoor, Azza I. Othman, Mohamed A. El-Missiry

**Affiliations:** 1 Department of Zoology, Faculty of Science, Mansoura University, Mansoura, Egypt; 2 Department of Medical Laboratory, Health Science, University of Gharyan, Gharyan, Libya

**Keywords:** Male infertility, Depakine, Testosterone, Testes, Androgen receptors

## Abstract

**Background:**

Depakine (valproic acid) is an antiepileptic medication that is commonly used as a first-line treatment for a variety of seizures in both adults and children. However, it can result in testicular toxicity by increasing oxidative stress inflammation. Melatonin (MLT) has antioxidant, anti-inflammatory, and anti-apoptotic potential. Therefore, the present study investigated the impact of MLT on depakine-induced testicular damage in rats.

**Methods:**

Four groups of male Wistar rats were formed, each of 5 animals: Group 1 was the control group; Group 2 was the MLT-treated group, receiving 20 mg MLT/kg BW; Group 3 was the depakine group, receiving 45 mg/kg; and Group 4 was the MLT + depakine treatment group, which received MLT and depakine for 14 days. Drug treatments were by gavage and daily.

**Results:**

Coadministration of MLT and depakine significantly (*P* < 0.001) improved the levels of testosterone and the expression of androgen receptors in the testes, explaining the improvement of sperm count, motility, and abnormalities. Similarly, spermatogenic cell depletion and shrinkage of seminiferous tubules were prevented by MLT + depakine treatment. Moreover, the testicles of rats given MLT + depakine had common histological architecture of seminiferous tubules, perimeter, and diameter, indicating melatonin’s anti-reproductive disruption. The combined treatment with MLT and dapakine resulted in the normalization of hematological parameters, including erythrocyte, platelet, and leukocyte counts; hematocrit content; mean cellular volume; mean cellular hemoglobin; and mean cellular hemoglobin concentration to levels comparable to the control group. These effects were associated with the enhancement of nuclear factor erythroid 2-related factor-2 and glutathione levels. Moreover, reactive oxygen species and malondialdehyde formation were decreased in the testis compared to the depakine-treated rats, indicating improvement in the redox status in the testis. The improvement of redox balance caused a remarkable regression of apoptotic regulating proteins (Bax, Bcl-2, and caspase-3) in the testis and downregulated inflammatory cytokines and chemokines (NF-κB, TNF-α, IL-6, ICAM-1, and MCP-1), indicating protection of spermatogenic cell viability.

**Conclusions:**

The combination treatment with MLT and depakine sustained male reproductive status, which can be attributable to the integrated antioxidant, anti-inflammatory, and antiapoptotic properties of MLT. These results may contribute to increase the clinical utility of depakine as a successful choice for neurological disorders.

## Introduction

Depakine, the brand name of valproic acid (VPA), is a well-known antiepileptic drug utilized clinically for the treatment of a variety of neurological disorders, including epileptic seizure and migraine. [1, 2]. However, treatment with depakine is associated with a number of adverse effects that can limit its clinical use. The main toxic effects of depakine include teratogenicity, nephrotoxicity, neurotoxicity, hepatotoxicity, and reproductive toxicity [1, 3]. Regarding reproductive toxicity, the treatment with valproate decreases male fertility, the expression of testicular proteins involved in spermatogenesis, and acrosome structure, decreasing testosterone synthesis in both epileptic men and experimental animals [4, 5]. It has been reported that depakine administration causes atrophy of the testis, epididymis, prostate gland, and seminal vesicles [6]. The testicular atrophy has been shown to be associated with adverse male reproductive and decreased sex hormone levels [7], which results in male infertility [8]. In rats, depakine alters the histology and down-regulates androgen receptors in the testis and epididymis [9]. It was reported that these side effects are mediated by oxidative stress, inflammation, and mitochondrial dysfunction [10].

Bone marrow is one of the most vulnerable and sensitive sites for drugs and harmful substances. Anemia may have an impact on reproductive health. Red blood cell disorders can disrupt male fertility and reproductive health due to iron imbalance, which leads to decreased gonadotropin release and sperm counts [11]. Direct suppression of the bone marrow activity by valproate can result in aplastic anemia or peripheral cytopenia that affects hematopoiesis [12, 13]. There is not enough evidence linking depakine to male fertility in terms of the hematopoietic system and possible treatment.

The nuclear factor erythroid-derived 2 (Nrf2) is a cellular response to intrinsic or extrinsic stresses that mediates antioxidant response. It has been observed that Nrf2 controls cellular resistance to oxidative stress, inflammation, and environmental toxins, which affects the physiology and pathology of testicular dysfunction, particularly the spermatogenic process [14]. Nrf2, with its coordinated genes, is widely expressed in the testis and plays an essential role in controlling the blood-testis barrier, testicular tissue functions, and defense against testicular toxic effects [14, 15]. Recently, it was reported that heat stress-induced Sertoli cell damage was prevented by Nrf2 activation in mice [16]. However, the impact of Nrf2 and antioxidants on male reproductive toxicity and potential protection after depakine treatment is insufficient.

The stimulation of oxidative stress and activation of inflammatory factors are thought to be significant causes of drug-induced toxicity to the testicles. A recent study demonstrated that treatment with valproate initiated oxidative damage, inflammatory, and apoptotic processes [17]. TNF-α and IL-1β levels in testicular tissue were highest in the valproic acid-treated rats [18]. The data from research involving acute testicular inflammation indicate elevation of several cytokines and adipokines, which has detrimental effects on germ cells and male fertility [19, 20].

Melatonin (MLT; N-acetyl-5-methoxytryptamine) is an important biological indolamine molecule synthesized from tryptophan via the serotonin pathway [21]. It is produced by various organs in all mammals, including humans, various animal taxa, microorganisms, and plants [22]. In addition to its circadian cycle regulation, MLT shows several biological functions, such as antioxidant, free radical scavenger, anti-inflammatory, and antiapoptotic impacts, and it can protect mitochondrial integrity and function [23]. It can easily cross biological membranes due to its high lipophilicity that enables it to reach high concentrations in tissue, cells, and subcellular compartments [24]. MLT has been shown to exert the protective activity by affecting miRNA expression [25, 26]. MLT can act through its two main types of receptors: G-protein-coupled receptors (MT1, MT2) that can initiate intracellular signal transduction cascades and the quinone reductase enzyme family (MT3), which is a detoxification enzyme [27]. Intake of a usual dose (i.e., 1 to 5 mg) within an hour after ingestion results in MLT concentrations 10 to 100 times higher than the physiological nocturnal peak, with a return to basal concentrations in 4 to 8 h [28].

MLT is present in the reproductive tract, seminal fluid, and spermatozoa. A significantly positive link was found between sperm motility and serum MLT levels [29]. In mammals, MLT regulates testicular blood flow and semen quality via the hypothalamic-pituitary–gonadal axis at various levels. This hormone stimulates steroidogenesis and spermatogenesis through MT1 and MT2 receptors, which is necessary for the function of Leydig cells, Sertoli cells, germ cells, and epididymal epithelial cells [30]. Additionally, MLT influences the Leydig cells, which are the primary source of testosterone [31] and protects the testicles and spermatogonia from oxidative damage exerted by various stressors and enhances the quality of the sperm [31, 32]. Recent findings on protective MLT functions in the field of male reproductive medicine have not received much attention. There are insufficient data on the effects of depakine on testicular toxicity when combined with MLT. There is a lack of evidence on the effect of concurrent treatment with MLT and depakine, triggering the need for more research. In order to gain precise insight into MLT's potential adjuvant role in protecting male reproductive health and increasing the use of depakine for neurological therapy, we investigated the effect of combined treatment with MLT and depakine on depakine-induced reproductive toxicity in male rats.

## Materials and methods

### Chemicals

Depakine (sodium valproate) (Sanofi, Global) was purchased from a local pharmacy. Melatonin (MLT) was provided by Sigma Chemical Company (St. Louis, MO, USA).

### Animals and experimental design

Male adult Wistar rats weighing 120 ± 5 g were obtained from the VACSERA, Cairo, Egypt. The rats were kept in plastic cages with daily replacements of wood-chip bedding. All rats were kept in a controlled environment with a temperature of 25 °C and a 12-h light/dark cycle. The animals had free access to water and a commercial rodent pellet. The Mansoura University Animal Care and Use Committee (MU-ACUC) approved all experimental protocols (approval number, MU-ACUC, SC.R.23.11.14).

**Fig. 1:**
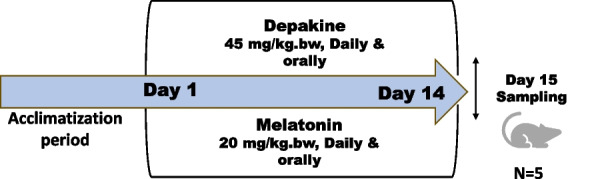
Schematic diagram of the experimental design. Rats were orally administered depakine and melatonin (MLT) for 14 days. At the end of the experimental period (14 days), overnight-fasted animals were euthanized and then sacrificed, followed by sample collection

After a week of acclimatization, rats were allocated to four groups of five rats each as follows: Group 1 (control group): Rats did not receive any treatment. Group 2 (melatonin-treated group) (MLT): Rats received 20 mg MLT/kg BW orally daily [33] for 14 days. Group 3 (Depakine group): Rats were orally administered a therapeutic dose of depakine at 45 mg/kg dissolved in water [34] for 14 days daily in the morning based on a previous study [9]. Rats in Group 4 (MLT + Depakine treated group) were given the same daily dosages of MLT and depakine for 14 days as those in groups 2 and 3 (Fig. 1).

### Sample collection

At the end of the experimental period (14 days), rats were fasted overnight and anesthetized with ketamine/xylazine at a dosage of 0.55 mL/100 g body weight intraperitoneally.

A heart puncture was used to obtain blood samples. For each rat, a portion of the blood sample was collected in a heparinized tube to perform a complete blood count (CBC). The other portion was collected in a nonheparinized tube to prepare serum by cool centrifugation at 1500 rpm for 15 min at 4 °C, and it was then stored at −20 °C until biochemical analysis.

The anesthetized rats were then decapitated, and testes and cauda epididymis were removed and cleansed with regular saline. Small, deep slits were made along each epididymis's proximal and distal cauda to release sperm. The sperm suspensions were then carefully combined and used directly for sperm analysis and morphometric studies after being incubated for five minutes at 37 °C to guarantee full sperm release. The left testes were homogenized in child Tris–HCl buffer (0.1M, pH 7.4), centrifuged at 1500 rpm, and the resultant supernatant was kept at −20 °C until biochemical analysis. For histological and immunohistochemical (IHC) studies, the right testes were fixed in neutral formalin (10%) for subsequent processing.

### Sperm analysis

After dilution with fresh phosphate-buffered saline (PBS, 1:20 dilution), sperm count and percent of dead sperm using eosin-nigrosin stains were measured using a hemocytometer in accordance with the previously described procedures [35, 36]. For the percentage of progressive motility (PM%), a fresh, undiluted sperm sample was examined, and the percentage of sperm exhibiting progressive motility was calculated using phase-contrast microscopy. To evaluate sperm abnormalities, including banana-shaped head and short tails, a sperm smear was stained with hematoxylin and eosin. An Olympus light microscope (Amscope MU1000) was used to analyze 300 sperm per smear at 1000 × magnification, and they were photographed using a camera.

### Histopathological study of testis sections

To assess histopathological changes, right testes were fixed, dehydrated, cleared, and then embedded in paraffin wax. Sections (4-μm) were stained with hematoxylin and eosin, then examined using an Olympus light microscope, and then photographed using an Amscope MU1000 camera. The morphometric valuation of seminiferous tubules was performed using a pre-calibrated measuring eyepiece, the diameter and perimeter of each section's seminiferous tubules (mostly from circular tubular cross sections). The sections were examined at × 100 and 400 (Olympus light microscope), and measurements were made using ImageJ software. Measuring the tube diameter was under × 400 magnification.

### Immunohistochemical study of androgen receptor (AR)

Xylene was used to deparaffinize the sections of testes for immunohistochemical staining with the labeled streptavidin–biotin immunoperoxidase [37]. Briefly, the sections were incubated overnight at 4 °C at a dilution of 1:20 using the rabbit polyclonal (anti-AR) primary antibody (Cat # PA1-110) from ThermoFisher Scientific, USA, in accordance with the manufacturer's instructions. The detection was performed using goat anti-rabbit HRP-conjugated secondary antibody (Cat. #A16110), ThermoFisher Scientific, USA, at a dilution of 1:500. The labeling index was quantified using ImageJ software.

### Hematological analysis

Complete blood cell counts (CBC) were performed using the blood cell counter Sysmex XP 300.

### Biochemical analysis

ELISA kits (Crystal Chem Inc., USA) were used to determine the amount of testosterone (Catalogue # 80,550) in serum, and the procedures were followed as described in the manufacturer's instructions.

Nuclear factor kappa B (NF-κB) (Catalogue # MBS859331), tumor necrosis factor alpha (TNF-α) (Catalogue # MBS590025), interleukin-6 (IL-6) (Catalogue # MBS269892), intercellular adhesion molecule-1 (ICAM-1) (Catalogue # MBS714244), and monocyte chemotactic protein-1 (MCP-1) (Catalogue# MBS631867) cytokine levels were estimated in serum following ELISA kits provided by MyBioSource (San Diego, CA, USA).

The concentration of malondialdehyde (MDA) (Catalog# MBS738685), reactive oxygen species (ROS) (Catalog# MBS2540517), and glutathione (GSH) (Catalog# MBS1600118) was estimated in testes homogenate following the manufacturer’s instructions of the ELISA kits purchased from MyBiosource (San Diego, USA). Rat nuclear factor erythroid 2-related factor 2 (Nrf2) determination was performed using the ELISA kit according to technical instructions provided by MyBiosource (MBS752046). The quantitative estimation of caspase-3 (Catalog# MBS743552), Bax (Catalog# MBS2512405), and BCL-2 (Catalog# MBS2515143) concentrations in the testis homogenates was determined using the ELISA technique following the instructions of the kits obtained from MyBiosource (San Diego, USA).

### Statistical analysis

The number of animals in each group was determined based on a G*Power analysis. The Shapiro–Wilk normality analysis indicated that the raw data were normally distributed.

All data values were presented as mean ± the standard error of the mean (SEM). Then statistical comparisons were made by one-way analysis of variance (ANOVA) followed by Duncan's multiple range tests as a post-hoc test for multiple comparisons using GraphPad Prism 8.0 software. Values are expressed as mean ± SEM (*n* = 5).

## Results

### MLT improved sperm parameters in depakine-treated rats

**Fig. 2:**
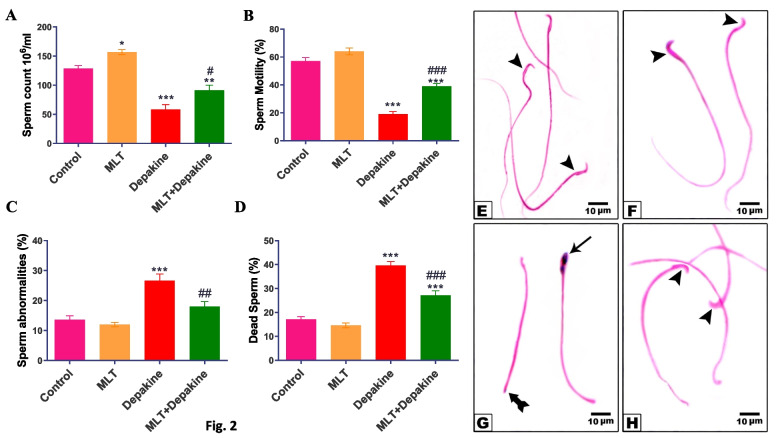
Effect of melatonin (MLT) and depakine on the sperm parameters including (**A**) sperm count, (**B**) sperm motility, (**C**) deformed sperms, and (**D**) dead sperms, in the control and various experimental animal groups. (**E–H**) Smears of sperm were stained to show tail and head abnormalities in the control and different animal groups, (**E** and** F**) control and MLT-treated groups, respectively, showing normal sperm morphology (arrowhead), (**G**) the depakine group showing banana-shaped head (**arrow**) and short tail (tailed arrow) sperm morphology, and (**H**) MLT + depakine-treated group with normal sperm morphology (arrowhead) as well after 14 days. (H&E), X 1000 Values are expressed as mean ± SEM (n = 5). *, # Significant at *P* < 0.05*.* **, ## Highly significant at *P* < 0.01*.* ***, ### Very Highly significant at *P* < 0.001*.* *, **, *** Significant as compared with the control group. #, ##, ### Significant as compared with the depakine group.

Oral depakine administration for 14 days resulted in a considerable (*P* = 0.0001) decrease in sperm motility and count (Fig. 2A-B) and a significant (*P* = 0.0001) increase in morphological sperm abnormalities, including banana-shaped head and short tails, and dead sperm percent when compared to the control group (Fig. 2C-D, G). However, the daily MLT administration to rats treated with depakine for 14 days resulted in a marked decrease in disruption of sperm parameters and showed normal sperm morphology as compared to the animals treated with depakine. MLT administration daily for 14 days enhanced sperm count (Fig. 2A).

### MLT improved histological changes in testes

**Fig. 3:**
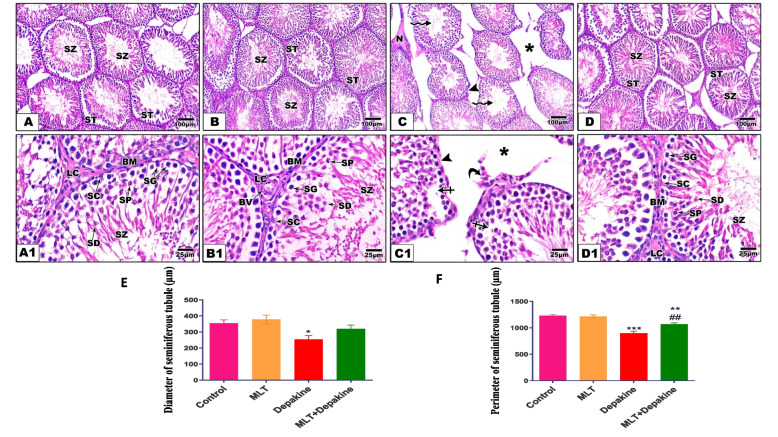
(**A-D**) Represent the impact of daily melatonin (MLT) and depakine treatment on histopathological alterations in representative images of testicular histology obtained at different magnifications in different rat groups. (**A** and **B**) Testicular section of the control and MLT-treated rats showing well-developed seminiferous tubules (ST) enclosed by an intact basement membrane (BM), regular arrangement of germinal epithelium spermatogonia (SG), Spermatocyte (SP), spermatids (SD), spermatozoa (SZ) filling the tubular lumen, Sertoli cell (SC), and prominent interstitial cellularity Leydig cell (LC). (**C**) Testicular sections of depakine treated rats illustrate irregular seminiferous tubule appearance with detached and distorted basement membranes (arrowhead), loss of cell homogeneity (crossed arrow), widened interstitial space (asterisk), necrosis of some areas (**N**), damaged and poorly developed Leydig Cell (curved arrow), and sperm debris in the lumen (zigzag arrow). (**D**) Concurrent treatment with MLT and depakine displayed amelioration of seminiferous tubule and spermatogenesis in most of the seminiferous tubules with minor cell debris. (H&E, X100 and 400 respectively). Quantification of the effect of MLT and depakine on the changes in the testicular morphology of seminiferous tubules after 14 days of treatment is expressed as the diameter and perimeter of seminiferous tubules (µm) in the studied groups (**E** and **F**, respectively). Each value is represented as mean ± SEM of 5 microscopic fields/tissue samples. * Significant at *P* < 0.05*.* **, ## Highly significant at *P* < 0.01*.* *** Very Highly significant at *P* < 0.001*.* *, **, *** Significant as compared with the control group. ## Significant as compared with the depakine group

The testicular histopathology for each experimental group was shown in Fig. 3. Testicular sections from the control and MLT-treated groups displayed ordinary seminiferous tubule architecture in addition to normally organized active spermatogenic cell layers and spermatozoa. On the other hand, depakine treatment for 14 days resulted in degenerative changes in the seminiferous tubules, including vacuolation of the seminiferous epithelium, necrosis, shrinkage, disordered and less compact tubule walls with detached basement membranes, and few spermatogenic cells. Treatment with depakine daily for 14 days resulted in a remarkable shrinking of seminiferous tubules with increased interstitial spaces. The depakine-induced histological change was significantly improved by the concurrent administration of MLT and depakine, allowing seminiferous tubules to restore both their architecture and cells of spermatogenesis (Fig. 3). The combined treatment (MLT + depakine) maintained the diameters and perimeters of the seminiferous tubule as well as normal interstitial spaces close to the control values compared with depakine-treated rats.

### MLT improved the expression of testicular androgen receptors (AR) and serum testosterone levels of depakine-treated rats

**Fig. 4:**
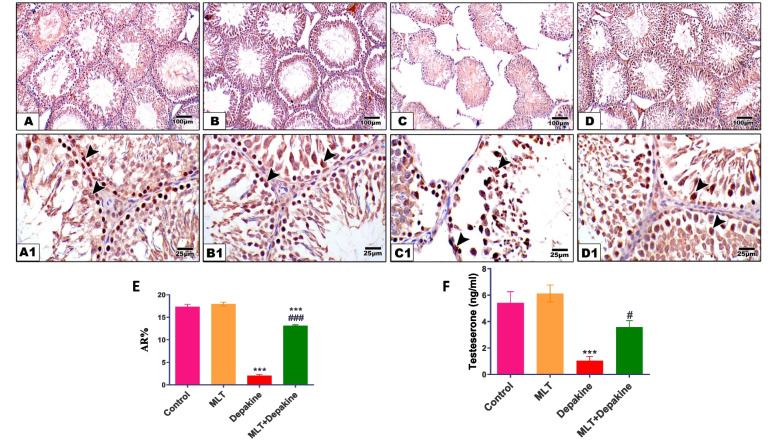
Impact of melatonin (M LT), depakine and their combination on immunohistochemical staining of androgen receptor (AR) in the testis and serum testosterone levels of the control and various treatment rats. The control (**A**) and MLT-treated rats (**B**) demonstrated strong AR immunostaining within germ cells (arrowheads). Testicular sections of (**C**) depakine-treated rats reveal mild AR expression within the different germ layers (arrowheads). (**D**) Concurrent treatment with melatonin + depakine displays a marked AR immuno-expression (arrowheads) within germ cells, (IHC × 100). (**E**) Quantification of the expression levels of AR in the control and different treatment groups. (**F**) Serum testosterone levels. Values are expressed as the means ± SEM of 5 microscopic fields/tissue samples. # Significant at *P* < 0.05*.* ***, ### Very Highly significant at *P* < 0.001*.* *** Significant as compared with the control group. #, ### Significant as compared with the depakine group

Immunohistochemistry analysis of testicular sections from rats treated with MLT and control groups revealed a normal AR immunoexpression within different seminiferous tubular cells (Fig. 4). Conversely, compared to the control group, rats treated with depakine exhibited a significant decrease (*P* = 0.0001) in AR expression in their testicular regions. Furthermore, the concurrent administration of MLT and depakine for 14 days demonstrated a significant increase (*P* = 0.0001) in AR immune expression in testes as compared to the depakine-treated group (Fig. 4).

The serum testosterone level was significantly (*P* = 0.0006) lower in rats given depakine than in the control group (Fig. 4). However, after receiving both MLT and depakine together, the serum testosterone levels normalized (*P* = 0.185) and approached that of the control groups. Testosterone level was increased by 242.7% in (MLT + Depakine) group compared to the dapakine treated animals.

### Improvement of hematological markers in rats received combined treatment of MLT and depakine

**Fig. 5:**
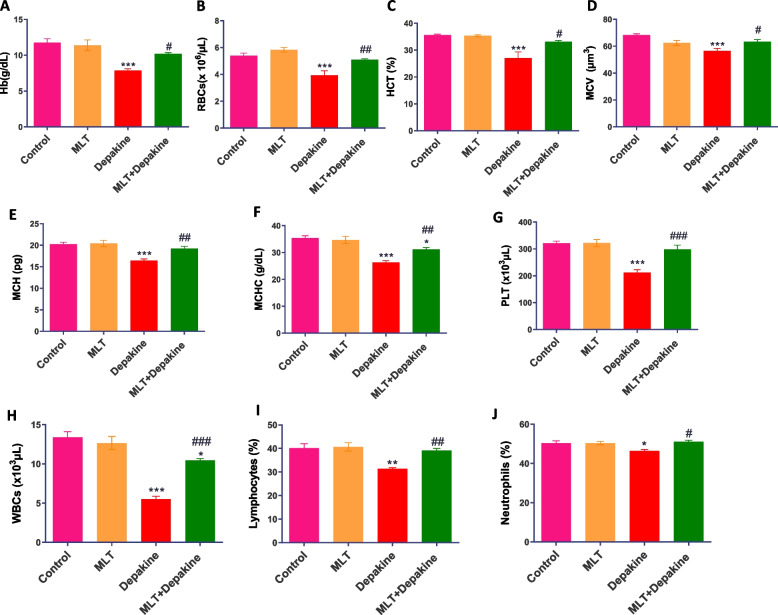
Impact of melatonin (MLT), depakine, and their combination on hematological parameters including (**A**) hemoglobin (Hb) content, (**B**) Red blood Cell count (RBCs), (**C**) hematocrit (HCT), (**D**) mean corpuscular volume (MCV), (**E**) mean corpuscular hemoglobin (MCH), (**F**) mean corpuscular hemoglobin concentration (MCHC), (**G**) platelets (PLT), (**H**) white Blood Cell (WBCs) count, (**I**) neutrophils, and (**J**) lymphocytes percentages in the control and different treated animal groups. Values are mean ± SEM (n = 5). *, # Significant at *P* < 0.05*.* **, ## Highly significant at *P* < 0.01*.* ***, ### Very Highly significant at *P* < 0.001*.* *, **, *** Significant as compared with the control group. #, ##, #### Significant as compared with the depakine group

Following 14 days of oral depakine treatment, the red blood cells (RBCs) count, hemoglobin (Hb) content, hematocrit (HCT) value, and the contents of mean corpuscular volume (MCV), mean corpuscular hemoglobin (MCH), and mean corpuscular hemoglobin concentration (MCHC) were significantly decreased compared with the control animals. Similarly, the counts of platelets (PLT), white blood cells (WBCs), neutrophils, and lymphocyte percentages were significantly lower in the depakine-treated rats than in the control group. Concurrent administration of MLT and depakine significantly ameliorated the impact of depakine on altered hematological parameters, resulting in levels that were comparable to those of the control groups (Fig. 5 A&B).

### MLT improved redox balance in depakine-treated rats

**Fig. 6:**
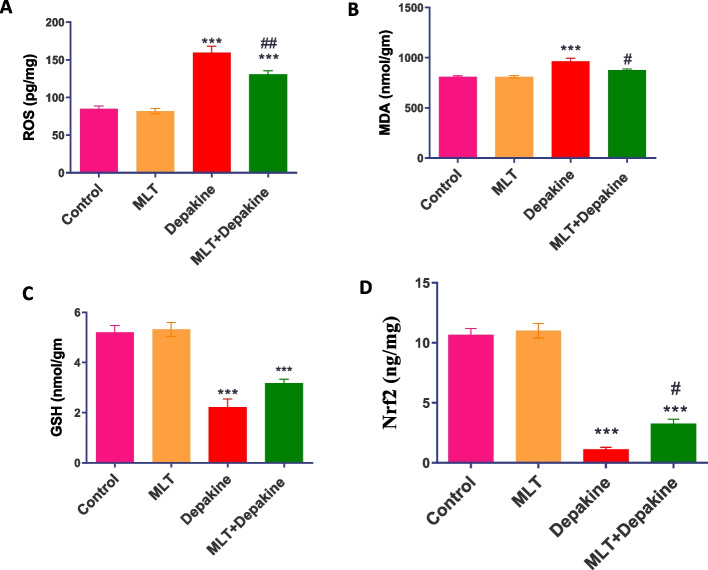
Impact of melatonin (M LT), depakine, and their combination on the levels of (**A**) reactive oxygen species (ROS), (**B**) malondialdehyde (MDA), (**C**) glutathione (GSH) and (**D**) nuclear factor erythroid 2 (Nrf2) in the testicular tissues of rats in the control and various treatment groups. Values are expressed as mean ± SEM (n = 5)**.** # Significant at *P* < 0.05*.* ## Highly significant at *P* < 0.01*.* ***Very Highly significant at *P* < 0.001*.* *** Significant as compared with the control group. #, ## Significant as compared with the depakine group

When compared to the control animals, the oral treatment of depakine for 14 days resulted in a significant (*P* = 0.0001) rise in oxidative stress markers, including ROS and MDA levels, while a significant decrease (*P* = 0.0001) in GSH and Nrf2 levels in the testes was observed. However, following a 14-day treatment of concurrent daily administration of MLT and depakine, the redox balance significantly improved, exhibiting a notable rise in Nrf2 and GSH levels as well as a decline in ROS and MDA accumulation to nearly normal values (Fig. 6).

### MLT ameliorated inflammatory mediators to normal levels in depakine-treated rats

**Fig. 7:**
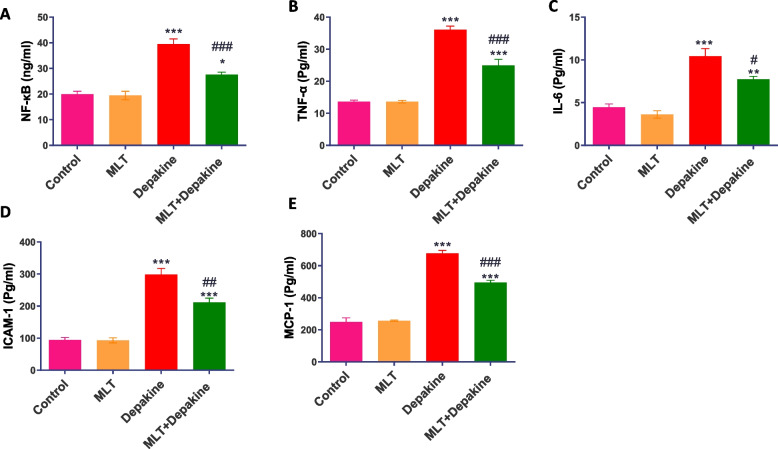
Impact of melatonin (MLT), depakine and their combination on serum levels of the inflammatory cytokines, (**A**) nuclear factor kappa B (NF-κB), (**B**) tumor necrosis factor alpha (TNF-α), (**C**) interleukin 6 (IL-6), (**D**) intercellular adhesion molecule-1 (ICAM-1), and (**E**) monocyte chemoattractant protein-1 (MCP-1), in the control and various treatment groups. Values are expressed as mean ± SEM (n = 5). *, # Significant at *P* < 0.05*.* **, ## Highly significant at *P* < 0.01*.* ***, ### Very Highly significant at *P* < 0.001*.* *, **, *** Significant as compared with the control group. #, ##, #### Significant as compared with the depakine group

Figure 5 illustrates the levels of inflammatory mediators in serum in the control and treated groups. The serum levels of NF-κB, TNF-α, IL-6, ICAM-1, and MCP-1 were significantly (*P* = 0.0001) higher in the depakine-treated animals than in the control group. MLT concurrently administered to mice treated with depakine for 14 days significantly ameliorated the upregulation of inflammatory cytokines in comparison to the animals treated with depakine (Fig. 7). Rats treated with MLT alone daily for 14 days did not affect the inflammatory parameters.

### MLT ameliorated changes in apoptosis-regulating protein levels in depakine-treated rats

**Fig. 8:**
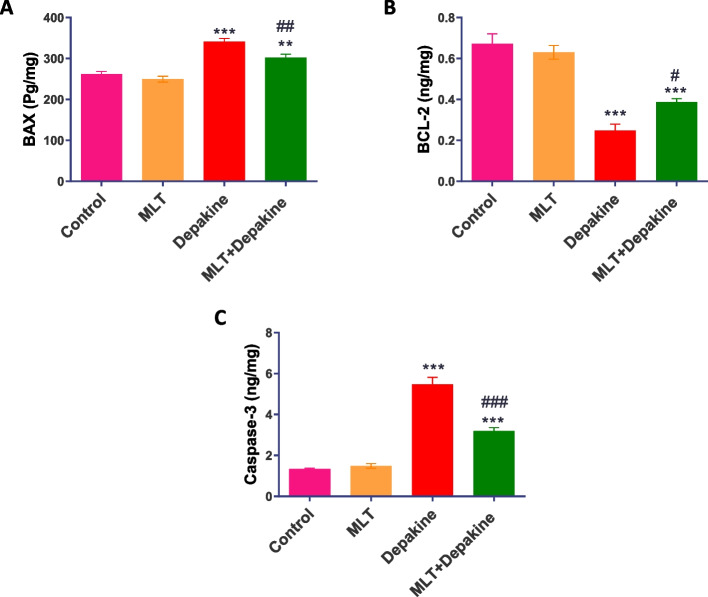
Effect of melatonin (M LT), depakine and their combination on the levels of apoptotic regulating proteins including (**A**) Bax, (**B**) Bcl-2, and (**C**) caspase-3 in the testes in various animal groups. Values are expressed as mean ± SEM (n = 5). # Significant at *P* < 0.05*.* **, ## Highly significant at *P* < 0.01*.* ***, ### Very Highly significant at *P* < 0.001*.* **, *** Significant as compared with the control group. #, ##, ### Significant as compared with the depakine group

Rats administered with depakine for 14 days demonstrated a significant (*P* = 0.0001) increase in pro-apoptotic proteins, including Bax and caspase-3, with a considerable decrease in the anti-apoptotic protein Bcl-2 when compared to the control group. In contrast, compared to the depakine-treated group, concurrent administration of MLT and depakine for 14 days markedly (*P* = 0.0001) improved the levels of these apoptotic regulatory proteins in testes to a comparable level to the control rats (Fig. 8). The levels of Bax, Bcl-2, and caspase-3 were not statistically changed in the testes of rats that were treated with MLT alone.

## Discussion

In the current study, MLT protected testicular structure and function, as well as sperm quality, in depakine-treated rats by improving hematological disruption, suppressing oxidative stress, reducing inflammation, and regulating apoptotic proteins, making it a promising pharmaceutical adjuvant for male reproductive health.

The anticonvulsant drug depakine is a beneficial medication for a variety of neurological disorders; however, testicular injuries have been documented [9, 38] and may induce male infertility [8]. The harmful effect of depakine on the testicles can be explained by a number of processes, including disruption of redox balance, inflammation, and apoptotic protein activation. Other pathways include hormonal disruption and epigenetic modifications. To maximize the clinical utility of depakine, we proposed it be taken in combination with an antioxidant adjuvant agent to overcome its oxidative toxicity and boost its efficacy. Multiple biological characteristics suggest MLT for therapeutic application for a variety of diseases and disorders for several reasons, including its ability to scavenge free radicals, function as an antioxidant, have anti-inflammatory impact, and anti-apoptotic potentials [39].

Hematological disorders can disrupt male fertility and reproductive health [11]. Significant alterations in hematological markers were observed in rats administered with depakine [40]. The most frequent hematological adverse impact was anemia, which was induced by valproate on DNA metabolism in bone marrow, which causes dyserythropoiesis. Depakine treatment had genotoxic and cytotoxic effects on red bone marrow and hepatocytes in mice [41]. The current investigation is in line with other research that demonstrated anemia as a side effect of sodium valproate therapy in adults and children of both sexes [42, 43].

The combined treatment with MLT and depakine maintained hematological parameters and indices within control values, indicating that MLT prevented the adverse effects of depakine on hematological parameters. The present findings are in line with previous data under various conditions. A previous study reported that the activity of MLT-induced opioids on κ-opioid receptors found on stromal bone marrow macrophages influences hematopoiesis, and MLT protected the genetic material of hematopoietic cells against oxidative damaging effects of acute total body irradiation [44] and lead-induced hematotoxicity [45]. MLT also alleviated the impact of the antidepressant fluoxetine-induced leukopenia, thrombocytopenia, and hypochromic and macrocytic anemia and improved the platelets and RBC indices [46]. Thus, the present data suggest that MLT may protect bone marrow and hematopoiesis during depakine treatment.

The present study showed that daily treatment with depakine resulted in testicular histopathological alteration, disruption of sperm parameters, low testosterone levels, and decreased expression of testicular androgen receptors (AR). These findings support earlier studies that treatment with valproic acid decreased the expression of AR in the testicles and epididymis and caused histopathological alterations in the germ cell epithelium in seminiferous tubules [9]. Although the precise mechanism by which depakine influences the quantity and quality of sperm is unknown, it has been shown that valproic acid inhibits the redox balance of mitochondria necessary for spermatozoa motility, which may result in sexual dysfunction [47].

The combination treatment with MLT and depakine enhanced sperm parameters, maintained testosterone levels within control value, and preserved the testicular histological structure. These findings suggest that MLT prevented the adverse effect of depakine on spermatogenesis by increasing the number, motility, and survival of sperm and improving their morphology. This might be related to the ability of MLT to improve the testicular oxidative state by increasing GSH and decreasing ROS and MDA levels in the testis, as shown in the current study. These findings support a prior study that found MLT raised total antioxidant capacity and lowered MDA and nitric oxide levels in the testes of MLT-treated rats under chemotherapy [48] confirming the importance of MLT in protection of reproduction [24]. The restoration of AR, sperm quality, and testosterone levels following 20 mg MLT/Kg treatment may indicate that MLT contributes to the protection of spermatogenesis and testosterone synthesis.

To explain the role of MLT in protection against testis toxicity, the current results showed a considerable up-regulation of AR expression in the testes of rats treated with a combination of MLT and depakine. Androgens and AR have a major impact on spermatogenesis and sperm parameters. The signals that testosterone provides through the AR in the testis are necessary for spermiation [31]. Since MLT is the primary physiological regulator of reproduction in mammals, a functional association between the pineal gland and the testis is proposed. Based on the fact that several testicular cells contain MLT receptors [31], it is most likely that MLT functions as an antioxidant and scavenges reactive oxygen species (ROS) to protect the expression of AR in the testis.

It has been reported that epileptic drugs, including depakine, caused redox imbalance with increased oxidative stress, which is the main damaging factor for organs [49]. Additionally, the testicular tissue is rich in polyunsaturated fatty acids [50, 51] which makes it more vulnerable to lipid peroxidation and membrane disruption by depakine. The current investigation demonstrated remarkably elevated oxidative stress in the testes of depakine-treated rats. On the other hand, MLT administration led to a considerable reduction in ROS in the testis of depakine-treated rats. This is in line with a study that demonstrated the notable ability of MLT to scavenge and lower ROS levels and prevent the depletion of natural antioxidant enzymes [52, 53]. Consequently, we observed that rats administered MLT had significantly less MDA level in their testicles, which suggests that the oxidative damage of testicular cells has decreased. The effectiveness of the antioxidant capacity of MLT is attributed to its amphiphilic nature, which allows it to cross biological membranes and reach cytosol, nucleus, and mitochondria [24], resulting in the protection of biological molecules of germ cells against ROS. Under oxidative stress, MLT can reduce the amount of ROS produced by the mitochondria in sperm [54] at an early point upon oxidative stress induced by H_2_O_2_ exposure [55]. Apart from its well-documented antioxidant and antiradical mechanisms, MLT chelates iron, which is involved in the Fenton/Haber–Weiss reactions. This minimizes the production of the extremely harmful hydroxyl radical and hydrogen peroxide, which in turn decrease oxidative stress [56]. This effect might explain the protection of the testis and sperm against depakine-induced oxidative stress and the improvement of sperm parameters in the current study.

Nuclear factor erythroid 2-related factor 2 (Nrf2) is associated with oxidative stress and a major signaling pathway to sustain physiological conditions by improving redox balance [57]. The antioxidant activity of MLT is mediated by Nrf2 [58]. The current study findings showed that MLT increases Nrf2 and inhibits oxidative stress induced by depakine in the testis by lowering ROS and malondialdehyde (MDA) levels and raising GSH levels. These results match earlier studies showing MLT can lower ROS levels, raise SOD activity and GSH content, and suppress the expression of TNF-a and inducible nitric oxide synthase in the testes of stressed mice [59]. Upregulation of Nrf2 by MLT was reported to increase the expression of the antioxidant enzyme heme oxygenase-1 [60]. Thus, it is suggested that MLT increases GSH synthesis via the enhancement of Nrf2 by replenishing the testes'GSH pool, which is essential for spermatogenesis and vital for male fertility [23]. This might preserve sperm quality and testis structure observed in the current study. This finding agrees with recent reports that GSH improved testicular morphology and sperm quality in rats treated with STZ [61] and Bisphenol-A [32].

Oxidative stress and inflammation are interconnected processes in various diseases and disorders. Since reduced levels of MLT in the testicles are associated with male infertility related to inflammation [62, 63], we hypothesized that MLT could be a therapeutic agent to improve the testicular microenvironment and prevent dysfunction related to inflammation and oxidative stress. In order to verify this hypothesis, the effect of concurrent MLT supplementation on the testes of rats treated with depakine was investigated by evaluating inflammatory mediators. In contrast to rats treated with depakine alone, the rats treated with MLT and depakine experienced a considerable reduction in the elevated levels of inflammatory cytokines and chemokines (NF-kB, TNF-α, IL-6, ICAM-1, and MCP-1), indicating the anti-inflammatory effect of MLT. These results verify previous work in which MLT improved antioxidant capacity and suppressed inflammatory response via Nrf2 and NF-κB pathways in methotrexate-induced testicular damage [64] and mitigated ionizing radiation-induced testicular injury by suppressing inflammatory response and oxidative stress in rats [65]. It is also explained that, through suppressing NF-κB translocation, MLT decreases the production of pro-inflammatory cytokines, ICAM-1, and many adhesion molecules [66]. MLT promotes the synthesis of the anti-inflammatory IL-10 both locally and systemically [65, 67]. These effects reinforced the anti-inflammatory impact of MLT.

It is of value to mention that MLT did not change the biological functions of the testes in control groups (Fig. 2– 8) indicating its safety at 20 mg/kg. Numerous characteristics of MLT indicate its potential applications in a range of situations. Exogenous MLT is highly biologically safe and can be administered at a range of dosages, including very high doses [68]. Doses ranging from 10 to 100 mg/kg are being suggested and tested for effectiveness in a range of conditions and ages [69].

As demonstrated in this study, depakine could induce testicular damage of seminiferous epithelium via the decreased level of AR, an effect that was ameliorated by MLT. It is assumed that depakine might affect the AR-dependent genes responsible for the translation of many proteins important for spermatogenesis and testosterone synthesis. The effect of MLT on gene expressions of such proteins needs to be further elucidated to clarify their relations to the decreased levels of AR protein expression. Moreover, the signaling pathways are an important aspect of the MLT protective effect to be used as a successful adjuvant with depakine therapy and need further investigation. Data on the effect of MLT on key genes in the Nrf2 signaling pathway is insufficient and requires extensive study. Future research is critical to understand the other molecular mechanisms and functions of MLT receptors in order to determine their ability to alleviate the adverse effects of anticonvulsant medications on reproduction.

In conclusion, MLT is able to preserve the testicular structure and function, as well as sperm quality, in depakine-treated rats. This positive effect could be attributed to its ability to suppress oxidative stress, reduce inflammation, and regulate apoptotic regulating proteins. Accordingly, MLT might be a potentially effective pharmaceutical adjuvant that can be utilized in clinical practice for the prevention of testicular oxido-inflammation damage and apoptosis induced by drugs, including anticonvulsant medication.

## Data Availability

All data generated or analyzed during this study are included in this published article.
